# Treatment outcomes of children and adolescents receiving drug-resistant TB treatment in a routine TB programme, Mumbai, India

**DOI:** 10.1371/journal.pone.0246639

**Published:** 2021-02-18

**Authors:** Shubhangi Dhakulkar, Mrinalini Das, Narendra Sutar, Vikas Oswal, Daksha Shah, Shilpa Ravi, Dipa Vengurlekar, Vijay Chavan, Lorraine Rebello, Augusto C. Meneguim, Aparna Iyer, Homa Mansoor, Stobdan Kalon, Shrikala Acharya, Gabriella Ferlazzo, Petros Isaakidis, Harshad P. Thakur

**Affiliations:** 1 National TB Elimination Programme, Mumbai, India; 2 Médecins Sans Frontières/Doctors Without Borders, Mumbai, India; 3 Tata Institute of Social Sciences, Mumbai, India; 4 Mumbai Districts AIDS Control Society, Mumbai, India; 5 Southern Africa Medical Unit, Médecins Sans Frontières, Cape Town, South Africa; 6 National Institute of Health and Family Welfare, New Delhi, India; The Foundation for Medical Research, INDIA

## Abstract

**Background:**

Childhood and adolescent drug-resistant TB (DR-TB) is one of the neglected infectious diseases. Limited evidence exists around programmatic outcomes of children and adolescents receiving DR-TB treatment. The study aimed to determine the final treatment outcomes, culture conversion rates and factors associated with unsuccessful treatment outcome in children and adolescents with DR-TB.

**Methods:**

This is a descriptive study including children (0–9 years) and adolescents (10–19 years) with DR-TB were who were initiated on ambulatory based treatment between January 2017-June 2018 in Shatabdi hospital, Mumbai, India where National TB elimination programme(NTEP) Mumbai collaborates with chest physicians and Médecins Sans Frontières(MSF) in providing comprehensive care to DR-TB patients. The patients with available end-of-treatment outcomes were included. The data was censored on February 2020.

**Result:**

A total of 268 patients were included; 16 (6%) of them were children (0–9 years). The median(min-max) age was 17(4–19) years and 192 (72%) were females. Majority (199, 74%) had pulmonary TB. Most (58%) had MDR-TB while 42% had fluoroquinolone-resistant TB. The median(IQR) duration of treatment (n = 239) was 24(10–25) months. Median(IQR) time for culture-conversion (n = 128) was 3(3–4) months. Of 268 patients, 166(62%) had successful end-of-treatment outcomes (cured-112; completed treatment-54). Children below 10 years had higher proportion of successful treatment outcomes (94% versus 60%) compared to adolescents. Patients with undernutrition [adjusted odds-ratio, aOR (95% Confidence Interval, 95%CI): 2.5 (1.3–4.8) or those with XDR-TB [aOR (95% CI): 4.3 (1.3–13.8)] had higher likelihood of having unsuccessful DR-TB treatment outcome.

**Conclusion:**

High proportion of successful treatment outcome was reported, better than global reports. Further, the nutritional support and routine treatment follow up should be strengthened. All oral short and long regimens including systematic use of new TB drugs (Bedaquiline and Delamanid) should be rapidly scaled up in routine TB programme, especially for the paediatric and adolescent population.

## Introduction

The global burden of childhood tuberculosis (TB) was 1.1 million in 2018 [1]. An estimated 25000 children have multidrug resistant tuberculosis (MDR-TB), according to mathematical modelling studies [2]. It is well known that there are gaps in global estimated and reported numbers of MDR-TB in children, thus indicating that childhood MDR-TB is one of the neglected diseases in the world.

Though children with MDR-TB have better treatment outcomes than adults [3, 4], many children struggle during the treatment due to limited availability of paediatric formulation, child-friendly laboratory services and occurrence of adverse events due to injectable [5]. In case of adolescents, another vulnerable group, they often report poor adherence during MDR-TB treatment [6, 7], and later poor treatment outcome. Thus, 2018 World Health Organisation (WHO) ‘Roadmap towards ending TB in children and adolescents’ have emphasized on improved strategies to tackle childhood and adolescent MDR-TB [8].

Systematic reviews have shown favourable treatment outcomes for children with MDR-TB and XDR-TB (extensively drug resistant TB) [3, 4, 9], though the reviews recommend more field studies in multiple settings to understand the treatment outcomes in children with drug resistant TB (DR-TB). A recent systematic review of tuberculosis in adolescents highlights the need of policy recommendations for this distinct age-group, as they are distributed within children and adults [10]. In addition, programmatic experiences of providing treatment to children and adolescents with DR-TB, from routine national TB programmes in lower and middle income countries are still understudied.

India has been providing conventional 20–22 months MDR-TB treatment under National TB Elimination Programme (NTEP) to patients including children and adolescents in routine programme settings [11]. Since 2018, the India NTEP is providing short regimen for MDR-TB patients. Childhood MDR-TB is one of the top priorities of the national strategic plans 2017–2025 [12], however limited evidence exists around programmatic experiences of children and adolescents with MDR-TB receiving treatment in national TB programme.

In Mumbai, NTEP Mumbai, Municipal Corporation of Greater Mumbai (MCGM), private chest physicians and Médecins Sans Frontières (MSF)–a nongovernmental organisation (NGO) has been providing comprehensive care (under public-private-partnership model) including diagnosis and treatment for patients with DR-TB including children and adolescents since 2016 [13]. To add to the body of evidence for children and adolescents with DR-TB, the current study is aimed to determine the final treatment outcomes, culture conversion rates and factors associated with unsuccessful treatment outcome in children and adolescents with DR-TB.

## Methods

### Study design

This is a descriptive study using routine programme data.

### Study setting

Mumbai- a city in state of Maharashtra, India, is one of the most populous cities with a population of 12.5 million [14]. In 2019, an estimated 10621 patients with DR-TB were diagnosed in Maharashtra [11]. Mumbai contributes 22% of TB cases reported in the state of Maharashtra [15]. The city is known as a TB and DR-TB hotspot of the country and has a high proportion of MDR-TB with fluoroquinolone resistance and advanced TB resistance profiles [16–18]. M-East Ward is one of the wards in Mumbai which has lowest development index and majority of the residents live in slums [19]. The government tertiary care hospital (Shatabdi hospital) in Govandi, M-East Ward offers treatment for all illnesses to the patients in and outside the M-East ward.

The national TB elimination programme (NTEP) in collaboration with private chest physician and Médecins Sans Frontières (MSF) under public-private-partnership provides comprehensive care to patients with DR-TB in the out-patient department (OPD) of a government hospital (Shatabdi hospital), Govandi, Mumbai, India. The diagnosis and treatment to DR-TB patients is provided in DR-TB OPD as per India Programmatic management of drug-resistant tuberculosis (PMDT) guidelines [20]. Patients with presumptive TB are evaluated for TB and Rifampicin-resistance with GeneXpert in Shatabdi TB laboratory. Once diagnosed with Rifampicin resistant TB, first Line probe assay (LPA) and then culture-drug susceptibility testing (C-DST) are carried out in accredited laboratory under NTEP.

Patients are enrolled for care and the individualised treatment is started for the patients based on GeneXpert and available LPA results. Counsellors provide elaborate information about the disease, treatment; follow up visits, common adverse events during treatment, infection control measures to patients and family members. For children and adolescents, caregivers and family members are informed about the disease and treatment regimen. Age-appropriate customised adherence counselling is provided to the children and adolescents. Regular feedback is received from patients and caregivers for improving the treatment and care. Once treatment is initiated, patients are followed at designated health posts near their residence. MSF supports NTEP in nine of 15 health posts (covering almost half of the M-East Ward geographical region) for providing direct clinical care and counselling activities. In other six health posts, the counselling services are provided by counsellors of academic institution, Tata Institute of Social Sciences (TISS). Both the group of counsellors use similar counselling guidelines for adults based on national guidelines, though adapted for children and adolescents. Contact tracing is carried out for each enrolled patient.

The treatment regimens were initially designed based on 2017 PMDT India guidelines [21] and modified after second LPA and/or CDST results are received. Information on demographic, clinical and laboratory information are recorded in individual patient files and routine TB programme databases (Nikshay: electronic TB monitoring system [22]; and customized Excel database). Routine monitoring of culture follow up is carried out after third month of treatment. In case patient misses a routine follow up appointment, phone calls by counsellor and home visits by community health worker are made to understand the reasons of missing appointments. Patients are encouraged for routine follow up during treatment. Serious adverse event episodes are reported to pharmacovigilance unit registered in NTEP, though few gaps exist in routine monitoring of adverse events. Access to new drugs, (Bedaquiline and Delamanid) for children and adolescents at the Shatabdi DR-TB OPD was limited until 2018, and the roll out of the injectable-based Short Course Regimen, for eligible patients, only started in early 2019.

### Study population and participants

All children (0–9 years) and adolescents (10–19 years) with DR-TB that initiated treatment in Shatabdi hospital, Mumbai, India during January 2017- June 2018 and had end-of-treatment outcome were included in the study. Patients, who received ‘regimen change’ as interim outcome, were followed and monitored during next treatment phase and their subsequent treatment outcome was included.

### Operational definitions

1. **DR-TB Resistance profiles:** Standard definitions of pre-extensively drug-resistant tuberculosis (Pre-XDR TB) and; extensively drug-resistant tuberculosis (XDR-TB) was used.
2. **Treatment outcomes:** Standard definitions for treatment outcomes [cured, completed, failed, died, lost to follow up (LTFU)] were used [20]. The patients who received ‘Regimen change’ as interim outcome were identified in programme database and their subsequent end of treatment outcome was noted. Successful treatment outcome included cured and completed treatment outcome while the rest was categorized as unsuccessful treatment outcome.

### Data management and analysis

The data from routine programme electronic databases (Nikshay [22] and Microsoft Excel) were exported into and analysed using STATA (version 15, StataCorp, College Station, Texas). The data was censored on February 2020 and the databases were accessed on 10 March 2020 to obtain the data used the study. Continuous variables among demographic and clinical characteristics such as age, treatment duration were described as median (Interquartile range, IQR). Categorical variables like sex, nutritional status (based on WHO BMI for age: z score for age 5–19 years [23]), previous TB episodes, TB resistance profile were described using proportions. Numbers and proportions were used to summarize the analytic output (treatment outcomes). Unadjusted and adjusted analysis (odds ratio, OR) was carried out to determine the association of demographic, clinical characteristics with unsuccessful treatment outcome. Kaplan Meier curve was used to describe the culture progression of the patients over time.

### Ethics

The study was based on review of routine programme records of patients enrolled for treatment in national TB programme (NTEP) India. The database was exported in an anonymous version (without patient identifiers) and later analysed for the study. All data was made fully anonymous before it was accessed for the study. The study received ethics approval from Ethics Review Board of Médecins Sans Frontières, Geneva, Switzerland (MSF ID #1928, dated 12 September 2019) and Institutional Review Board of Tata Institute of Social Sciences, Mumbai, India (Serial No. 2018-19/19, dated 19 June 2019).

## Results

### Patient characteristics

A total of 268 patients were included in the study period. Of 268, the proportion of children (0–9 years) was 6% (Table 1). The median (min-max) age was 17(4–19) years. About 192(72%) patients were females and female to male ratio was 2.6:1. Among 203 patients aged 5–19 years (with available weight and height data), 80 (40%) were undernourished (19% moderate; 21% severely undernourished). One was co-infected with HIV.

**Table 1:** Demographic and clinical characteristics of children and adolescents with DR-TB who received treatment in government hospital, Mumbai, India, January 2017- February 2020, N = 268.

Characteristic	Number	Percentage
**Total**	**268**	**100**
**Age group (in years)**		
0–9	16	6
10–19	252	94
Age (Median, Min-Max, years)	17 (4–19)	
**Sex**		
Male	76	28
Female	192	72
Nutritional status* **(n-203)**		
Normal	123	60
Moderately undernourished	38	19
Severely undernourished	42	21
**HIV status**		
Positive	1	<1
**TB site**		
Pulmonary	199	74
Extra-pulmonary	69	26
**TB diagnosis**		
Bacteriological confirmation	265	99
Clinically diagnosed	3	1
**TB resistance profile**		
MDR-TB	155	58
Pre-XDR TB	78	29
XDR TB	35	13
**Previous episode of TB (n-257)**		
Yes	107	42
No	150	58
**Treatment regimen**		
Includes injectable	265	99
No injectable	3	1

*Nutritional status assessment was based on WHO BMI for age (z score) for 5–19 years (Patients with available information were included). MDR-TB: Multi drug resistant TB; Pre-XDR TB: Pre-extensively resistant TB (77 = Rifampicin +INH+ fluoroquinolone resistant; 1 = Rifampicin+INH+second line injectable resistant); XDR-TB: Extensively resistant TB

Majority (74%) had pulmonary TB and most of them (99%) had bacteriologically confirmed DR-TB (diagnosed by GeneXpert, LPA or C-DST). The most common site for extra-pulmonary TB (EPTB) was lymph node involvement (cervical, axillary and inguinal). The proportions of MDR-TB: Pre-XDR-TB: XDR-TB in patients was 58:29:13 and 42% patients had fluoroquinolone resistance. About 56% patients (n = 257) did not have history of previous TB episode.

Most (99%) patients received DR-TB treatment regimen including injectable. None received short course regimen or new TB drugs (Bedaquiline or Delamanid) in treatment regimen for DR-TB. The median (IQR) duration of treatment (n = 239) was 24(10–25) months.

### Culture conversion

**Fig 1:**
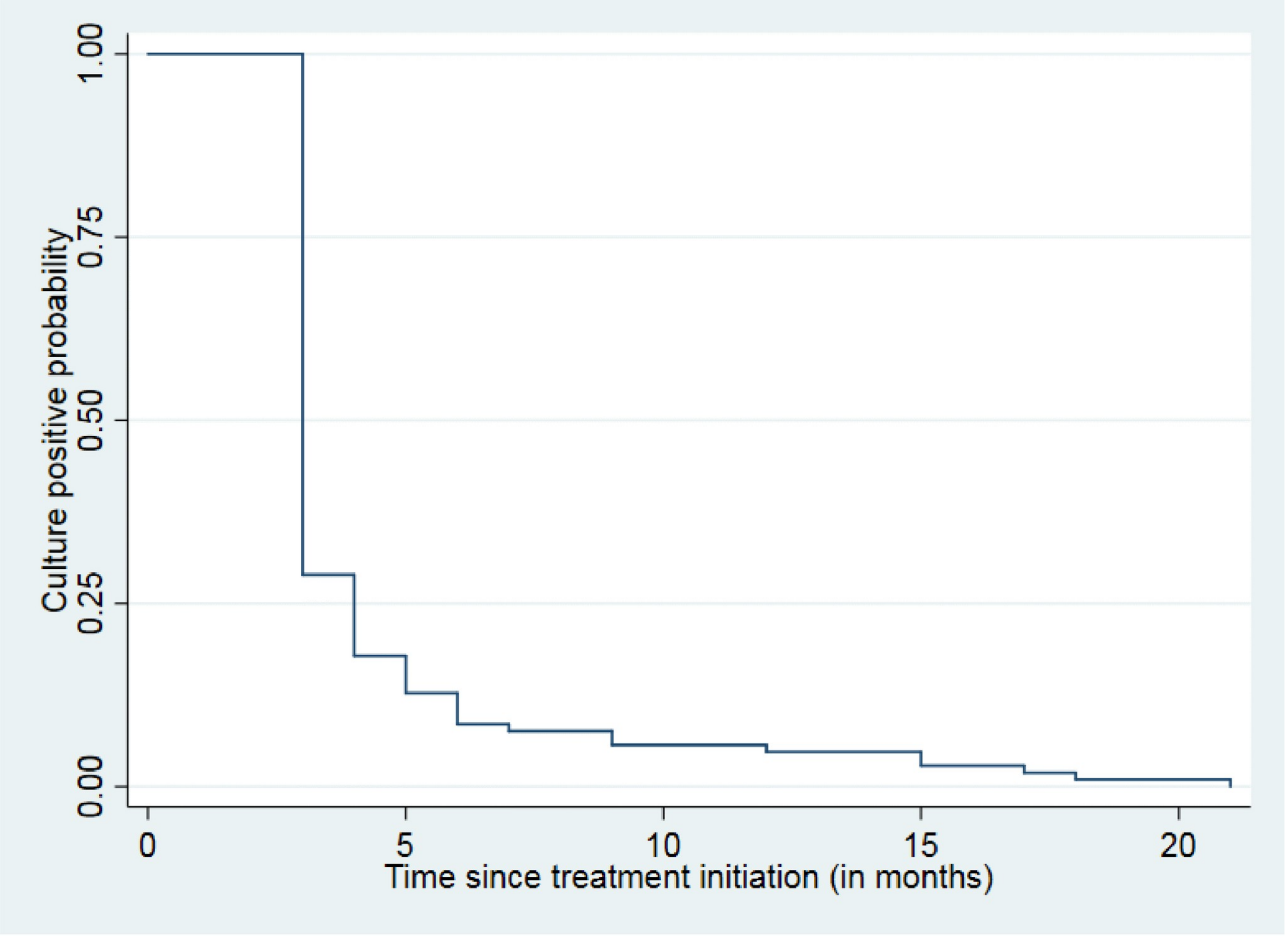
Culture positive probability from time of DR-TB treatment initiation in children and adolescents (culture positive at baseline and with available culture follow up results) who received DR-TB treatment in government hospital, Mumbai, India, January 2017- February 2020, N = 128.

A total of 180 children and adolescents (67% of 268) had positive baseline TB culture report. The follow up culture reports were available for 128 patients. Of these 128 patients, 94(73%) had culture conversion at third month and 113 (88%) by end of six months after treatment initiation. Though the proportions of available follow up culture reports were higher in females (73% versus 68%); less females had six month culture conversion than males (87% versus 92%). The median (IQR) time for culture-conversion was 3(3–4) months. Table 2 and Fig 1 shows the Kaplan Meier curve of time to culture conversion after treatment initiation (n = 128).

**Table 2:** Culture positive probability from time of DR-TB treatment initiation in children and adolescents (culture positive at baseline and with available culture follow up results) who received DR-TB treatment in government hospital, Mumbai, India, January 2017- February 2020, N = 128.

**Months from treatment initiation (Interval)**	0	3–4	4–5	5–6	6–7	7–8	9–10	12–13	15–16	17–18	18–19	21–22
**Number of patients at risk (n)**	128	128	34	21	15	9	8	6	5	3	2	1
**Proportion remaining culture positive (95% CI)**	100	0.27 (0.19–0.34)	0.16 (0.11–0.23)	0.12 (0.07–0.18)	0.07 (0.03–0.12)	0.06 (0.03–0.11)	0.05 (0.02–0.09)	0.04 (0.01–0.08)	0.02 (0.01–0.06)	0.02 (0.003–0.05)	0.01 (0.001–0.04)	0

### Treatment outcome

Of 268 patients, 166 (62%) had successful end of treatment outcomes including 112 (42%) who were cured while 54 (20%) completed treatment (Table 3). Among the rest, 44 (16%) died, 53(20%) were LTFU, and 5(2%) failed treatment.

**Table 3:** Treatment outcomes of children and adolescents with DR-TB who received treatment in government hospital, Mumbai, India, January 2017- February 2020, N = 268.

Treatment outcome	Total		Children		Adolescents	
	(0–19 years)		(0–9 years)		(10–19 years)	
	Number	(%)	Number	(%)	Number	(%)
**Total**	**268**	**(100)**	**16**	**(100)**	**252**	**(100)**
**Successful**	**166**	**(62)**	**15**	**(94)**	**151**	**(60)**
Cured	112	(42)	4	(25)	108	(43)
Treatment completed	54	(20)	11	(69)	43	(17)
**Unsuccessful**	**102**	**(38)**	**1**	**(6)**	**101**	**(40)**
Died	44	(16)	-		44	(17)
Lost to follow up	53	(20)	1	(6)	52	(21)
Failed	5	(2)	-		5	(2)

Overall males had higher proportion of unsuccessful outcome than female patients; however the occurrence of death were higher in females compared to males (3.4:1). On segregating pulmonary and EPTB cases, the proportion of treatment completion was higher in EPTB patients than pulmonary TB patients (42% versus 13%). Further, the EPTB patients had lower occurrence of death (3% versus 21%), failure (Nil versus 2.5%) and LTFU (16% versus 21%) compared to pulmonary TB patients.

On comparing the end of treatment outcome between children and adolescents with DR-TB (Table 3), children (n = 16) had higher proportion of successful end of treatment outcomes (94% versus 60%) compared to adolescents (n = 252).

### Factors associated with unsuccessful outcome

On assessing the association of select demographic and clinical characteristics with unsuccessful outcome (Table 4), the study identified that adolescent age group [OR (95% CI): 10.0 (1.3–77.1)], undernutrition [OR (95% CI): 3.8 (2.1–6.9)], pulmonary TB site [OR (95% CI): 3.5 (1.8–6.8)], advanced TB resistance profiles [Pre-XDR TB, OR (95% CI): 1.9 (1.1–3.4); XDR-TB, OR (95% CI): 7.5 (3.2–17.3)] and previous TB episode [OR (95% CI): 2.0 (1.2–3.4)] had higher odds of developing unsuccessful outcome. In adjusted analysis, patients with either undernutrition [aOR (95% CI): 2.5 (1.3–4.8)] or who had XDR-TB [aOR (95% CI): 4.3 (1.3–13.8)] had higher odds of having unsuccessful TB treatment outcome.

**Table 4:** Factors associated with unsuccessful end of treatment outcome among children and adolescents with DR-TB who received treatment in government hospital, Mumbai, India, January 2017-February 2020 (N = 268).

Characteristics	Unsuccessful outcome	Successful outcome	Unadjusted OR (95% CI)	Adjusted OR (95% CI)
**Total**	102 (38)	166 (62)		
**Age (In years)**				
0–9	1 (94)	15 (6)	1	1
10–19	101 (40)	151 (60)	**10.0(1.3–77.1)**	4.4 (0.4–42.9)
**Sex**				
Male	33 (43)	43 (57)	1.4 (0.8–2.3)	1.2 (0.6–2.4)
Female	69 (36)	123 (64)	1	1
Nutritional status*				
Undernourished	44 (55)	36 (45)	**3.8 (2.1–6.9)**	**2.5 (1.3–4.8)**
Normal	30 (24)	93 (76)	1	1
**TB site**				
Pulmonary	89 (45)	110 (55)	**3.5 (1.8–6.8)**	1.9 (0.8–4.4)
Extra pulmonary	13 (19)	56 (81)	1	1
**TB resistance pattern**				
MDR-TB	43 (28)	112 (72)	1	1
Pre-XDR TB	33 (42)	45 (58)	**1.9 (1.1–3.4)**	1.7 (0.8–3.4)
XDR-TB	26 (74)	9 (26)	**7.5 (3.2–17.3)**	**4.3 (1.3–13.8)**
**Previous TB episode**				
Present	48 (45)	59 (55)	**2.0 (1.2–3.4)**	1.4 (0.7–2.9)
Absent	43 (29)	107 (71)	1	1

*Nutritional status assessment was based on WHO BMI for age (z score) for 5–19 years. MDR-TB: Multi drug resistant TB; Pre-XDR TB: Pre-extensively resistant TB (77 = Rifampicin+INH+fluoroquinolone resistant; 1 = Rifampicin+INH+second line injectable resistant); XDR-TB: Extensively resistant TB; OR: Odds ratio

## Discussion

The majority (62%) of children and adolescents with drug-resistant TB who received ambulatory treatment in a government hospital in Mumbai, India were successfully treated, which was better than the reports at global level [1]. The proportion of successful outcome in children was high (94% versus 60%) compared to adolescents. Significant proportions of patients either died or were lost to follow up, who would have needed optimised treatment strategies including access to newer TB drugs (Bedaquiline or Delamanid) to reduce the risk of unsuccessful outcomes. Undernutrition and complex resistance profile (XDR-TB) were likely associated with unsuccessful outcome.

The patients had a low proportion (6%) of children (0–9 years) with DR-TB during study period, thus the results are to be mostly attributed to adolescents (10–19 years). The low numbers of younger children in routine TB programme highlights the known challenges in childhood DR-TB diagnosis and linkage to treatment [3]. The national TB programmes must identify novel ideas for case finding and supporting the families during diagnosis and treatment of paediatric patients with DR-TB [24], one such being using GeneXpert as a primary tool for TB diagnosis in children [25].

More than two-thirds of the patients were females. Males had worse DR-TB treatment outcomes; however the occurrence of death was more common in females (3.4:1) than males. There is a possibility that the females may be reaching the hospital late, in advanced stage of illness. Thus, the TB programmes must collaborate with maternal and child health programme and adolescents programme for early identification and regular follow up during TB treatment for both males and females.

Four of every ten patients were either moderately or severely undernourished. Undernutrition was also identified as a risk factor for unsuccessful TB treatment outcome, in line with previous studies [26, 27]. Nutritional programmes linkages with TB programme will help in early identification of undernourished patients and providing them nutritional support during TB treatment. The local community based organisations may also play a role in providing nutritional support and regular monitoring of nutritional levels in these vulnerable groups.

Most of the patients (99%) had bacterial confirmation of DR-TB diagnosis. Often, TB diagnosis in the younger children is done empirically on clinical ground due to lack of point of child-friendly and sensitive diagnostic tools [28, 29], and to the pauci-bacillary disease often observed in paediatric cases. The low proportion of clinically diagnosed DR-TB cases in this study could then be attributed to the low proportion of children in the study.

Almost all patients received injectable during the treatment. The adverse events related to injection use have been well documented [30, 31], and these drugs are not recommended anymore by WHO for use in DR-TB, with exception of amikacin and streptomycin if no other options are available [32]. Furthermore, there is a strong global movement towards rapid implementation of all oral regimens for all, especially paediatric and adolescent age groups. Two positive steps in this direction are WHO ‘Roadmap towards ending TB for children and adolescents’-2018 and recent WHO DR-TB guidelines- 2019 that mention about an important shift from injectable based to all oral regimens for children [8, 33]. The national TB programmes should rapidly roll out all oral regimens for DR-TB treatment and monitor the implementation at field level (including stock management, patient counselling and routine monitoring).

A quarter of patients, who had baseline positive culture for TB, had no records of follow up culture. This highlights the need of strengthening of routine monitoring system [12]. In addition, about one-fourth of the patients (with baseline positive culture and available follow up culture reports) were still culture positive at third month. The national programme may consider routine monitoring of culture from first month of treatment. Considering the turn-around-time for CDST of three-six weeks [34], the failure cases could be identified early and the regimen could be adapted.

The proportion of unsuccessful treatment outcome in children was lower than those in adolescents. Only one paediatric patient (6%) was LTFU while 20% of adolescent patients were LTFU. This is in line with previous studies highlighting promising treatment outcomes in children compared to adults [3, 4] and poor adherence and treatment outcomes in adolescents [7, 35, 36]. The national TB programmes should implement innovative strategies focusing adherence support in adolescents [10]. Since the adolescents are reported in either child (10–14 years) or adult cohorts (15 years and above), we recommend separate monitoring for adolescents in TB programme.

Three fourth of patients with XDR-TB had unsuccessful treatment outcomes and patients with XDR-TB were also identified as a risk for unsuccessful treatment. This is in line with the findings of systematic review by Osman et al. in 2019 and other studies [9, 37, 38]. The national TB programmes should consider new TB drugs (Bedaquiline or Delamanid or both) based treatment regimen for these patients, for improved DR-TB treatment outcome [39, 40]. With the rising levels of fluoroquinolone resistance in known DR-TB hotspots, it will be difficult to treat similar patients affectively without the use of new TB drugs.

Our study reports the national TB programme implementation in one of the urban slum DR-TB hotspots in the country, thus the results provide insights for similar high TB burden slum settings. The study had few limitations: individual adherence reports were not available for most of the patients in the customized electronic database. The study does not include reports on serious adverse events, as they were not routinely monitored in the routine programme database. The adverse events and interruption of individual drugs were mentioned in the patient treatment cards and only modification of the treatment regimen was mentioned in the database. Thus, it was difficult to understand the reason of modification, if it was due to LPA or CDST reports or occurrence of serious adverse events. However, in recent times, the TB programme has improved the monitoring of adherence and adverse events of the patients.

## Conclusion

A high proportion of successful treatment outcome was reported. Further, nutritional support and routine treatment follow up for children and adolescents needs strengthening. All oral long and short regimens, including systematic use of newer TB drugs (Bedaquiline and Delamanid) at ambulatory level must rapidly become accessible to all, especially the paediatric and adolescent patients.
